# Supplementary material for: Cohesin composition and dosage independently affect early development in zebrafish
Source: Development. 2024 Aug 1;151(15):dev202593. doi: 10.1242/dev.202593 (PMC11317101; doi:10.1242/dev.202593)
Supplement: Supplementary information [file develop-151-202593-s1.pdf]

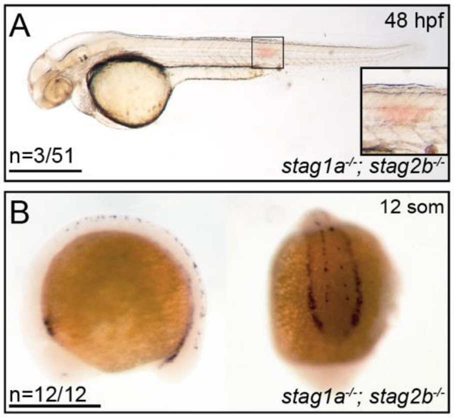

Fig. S1. Trunk hemorrhaging and *runx1* expression in *stag1a$^{-/-}$; stag2b$^{-/-}$* mutant embryos.

(A) Lateral views of representative and *stag1a$^{-/-}$; stag2b$^{-/-}$* embryos at 48 hpf. The boxed region (inset) outlines a trunk hemorrhage representative of those observed in around 5% of the *stag1a$^{-/-}$; stag2b$^{-/-}$* double mutant embryos. Scale bars are 500 µm.

(B) Normal expression of *runx1* at 12 somites in *stag1a$^{-/-}$*; *stag2b$^{-/-}$* embryos. Lateral (left) and posterior (right) views are shown. Scale bars are 500 µm. The numbers in the lower left hand corner indicate the number of embryos with the expression pattern shown.

Development • Supplementary information

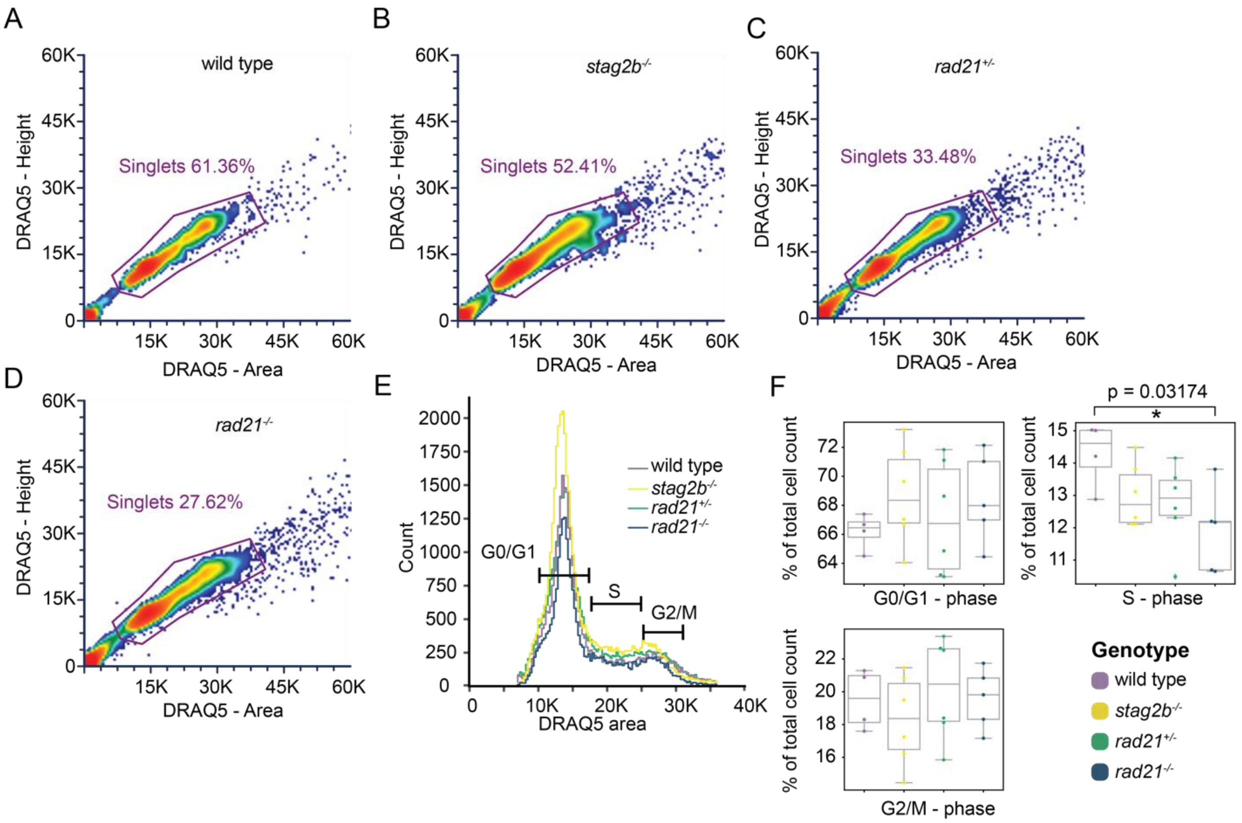

Fig. S2. Flow cytometry analyses show that *rad21*$^{-/-}$ tailbuds have fewer cells in S phase, while other cohesin-mutant genotypes resemble wild type. (A-D) Pseudocolour dot plot of flow cytometry dataset showing density of cells across DNA content shown as area (*x*-axis) and height of the signal (*y*-axis) of DRAQ5 (labeling DNA) for wild-type (A), *stag2b*$^{-/-}$ (B), *rad21*$^{+/-}$ (C) and *rad21*$^{-/-}$ (D) tailbud cells. **(E)** Count density plot of wild-type (purple), *stag2b*$^{-/-}$ (yellow), *rad21*$^{+/-}$ (green) and *rad21*$^{-/-}$ (blue) combined replicate samples (n>4). **(F)** Box and swarm plots showing the proportions of cell cycle phases as G0/1, S and G2/M based on DNA content. Boxes show quartiles, whiskers show 1.5 inter-quartile ranges of the lower and upper quartile. *P < 0.05 Mann-Whitney U test.

Development • Supplementary information

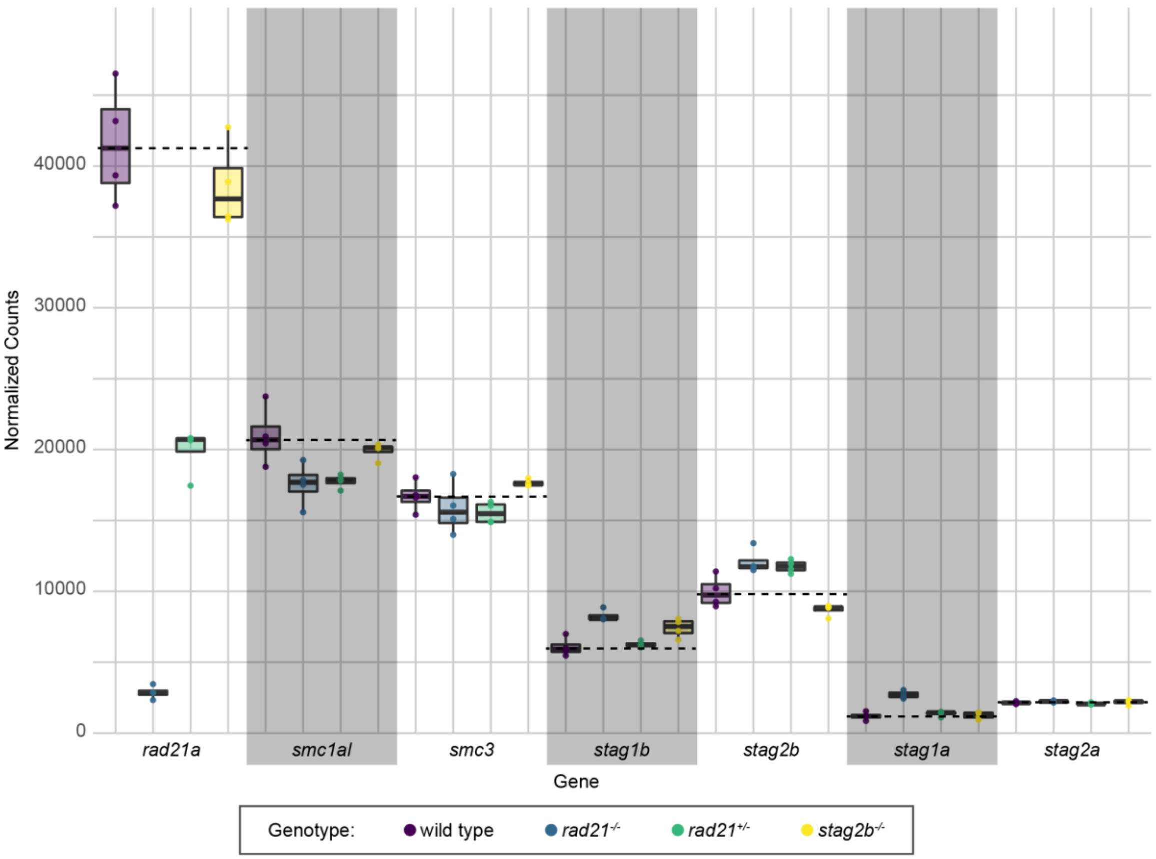

Fig. S3. Transcript counts of cohesin subunits in the tailbud.

Normalized transcript counts of *rad21a*, *smc1al*, *smc3*, *stag1b*, *stag2b*, *stag1a* and *stag2a* taken from RNA-seq of tailbuds and visualized with box plots defining the distribution of expression levels across these genes in the samples. Genotypes are distinguished by color: wild-type samples are displayed in purple, *rad21*$^{-/-}$ in blue, *rad21*$^{+/-}$ in green, and *stag2b*$^{-/-}$ samples in yellow. For each panel, the dotted line indicates the level of expression in wild type.

Development • Supplementary information

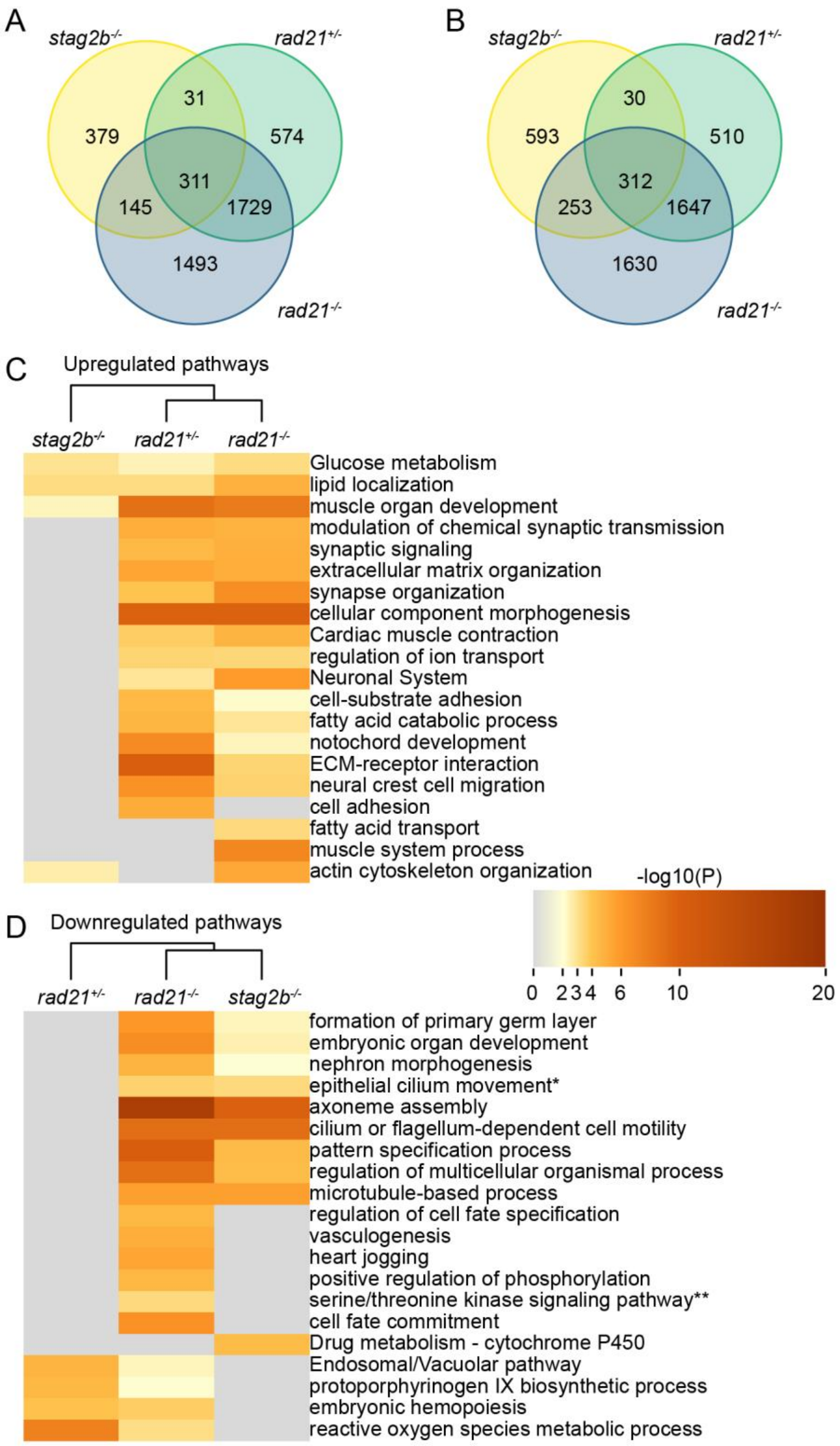
A
stag2b-/-
rad21+/-
rad21-/-
379
31
574
311
145
1729
1493
B
stag2b-/-
rad21+/-
rad21-/-
593
30
510
312
253
1647
1630
C
Upregulated pathways
stag2b-/-
rad21+/-
rad21-/-
Glucose metabolism
lipid localization
muscle organ development
modulation of chemical synaptic transmission
synaptic signaling
extracellular matrix organization
synapse organization
cellular component morphogenesis
Cardiac muscle contraction
regulation of ion transport
Neuronal System
cell-substrate adhesion
fatty acid catabolic process
notochord development
ECM-receptor interaction
neural crest cell migration
cell adhesion
fatty acid transport
muscle system process
actin cytoskeleton organization
-log10(P)
0
2
3
4
6
10
20
D
Downregulated pathways
rad21+/-
rad21-/-
stag2b-/-
formation of primary germ layer
embryonic organ development
nephron morphogenesis
epithelial cilium movement*
axoneme assembly
cilium or flagellum-dependent cell motility
pattern specification process
regulation of multicellular organismal process
microtubule-based process
regulation of cell fate specification
vasculogenesis
heart jogging
positive regulation of phosphorylation
serine/threonine kinase signaling pathway**
cell fate commitment
Drug metabolism - cytochrome P450
Endosomal/Vacuolar pathway
protoporphyrinogen IX biosynthetic process
embryonic hemopoiesis
reactive oxygen species metabolic process

Fig. S4. Overlap of dysregulated genes and pathway enrichment in cohesin mutant tailbuds.

(A, B) The Venn diagrams depict the overlap of significantly upregulated (A) and downregulated (B) genes in cohesin-deficient tailbuds. **(C, D)** Metascape heat maps displaying the top 20 terms enriched among significantly upregulated (C) and downregulated (D) genes in cohesin-deficient tailbuds. Corresponding *p*-values are indicated by the color scale.

*Epithelial cilium movement involved in extracellular fluid movement

**Regulation of transmembrane receptor protein serine/threonine kinase signalling pathway

Development • Supplementary information

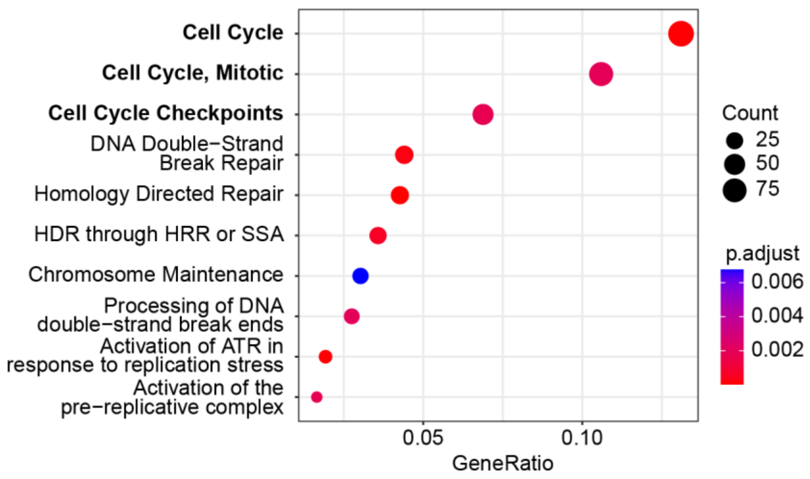

Fig. S5. Reactome analyses of downregulated pathways in *rad21*$^{-/-}$ mutant tailbuds.

The dot plot shows the top 10 enriched Reactome pathways (out of 26) among the significantly downregulated genes in *rad21*$^{-/-}$ tailbuds. The size of each dot indicates the number of genes affected in the pathway, and the dot color represents the adjusted p-value (Padj). Source data available in Tables S1-S3.

Development • Supplementary information

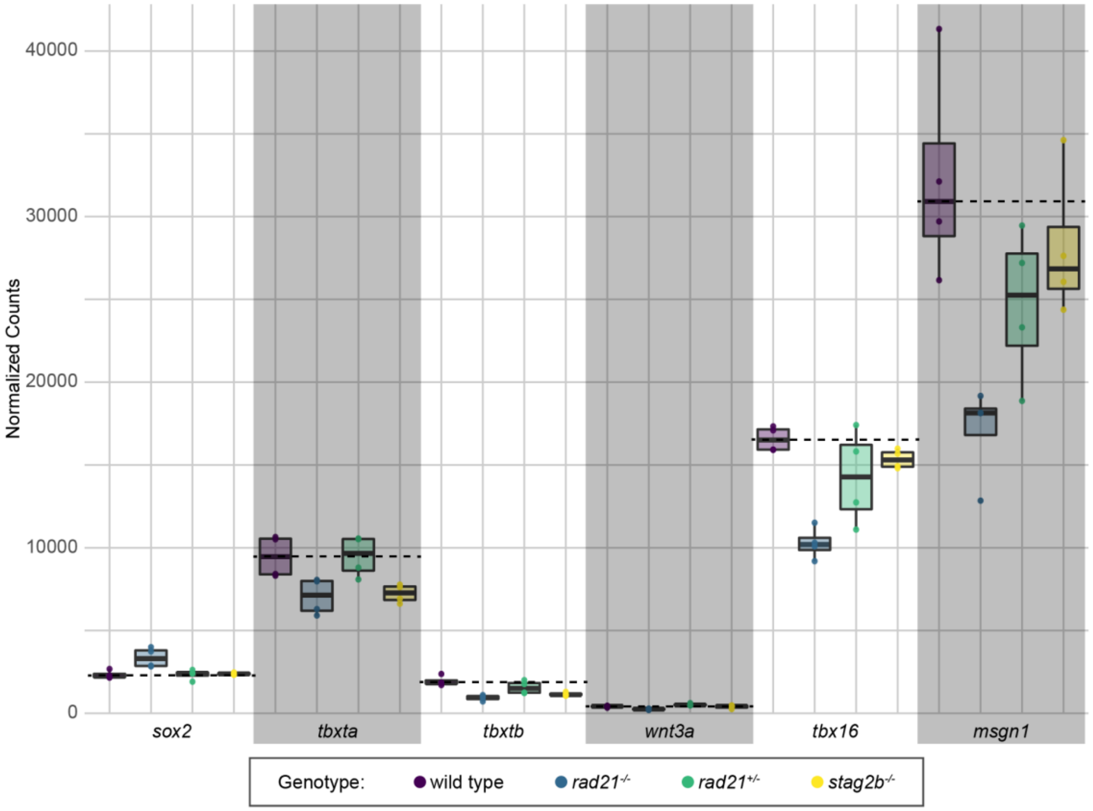

Fig. S6. Expression levels of *sox2, tbxta, tbxtb, wnt3a, tbx16* and *msgn1* in cohesin mutants. Normalized transcript counts of *sox2*, *tbxta*, *tbxtb*, *wnt3a*, *tbx16* and *msgn1* taken from tailbud RNA-seq data with 4 replicates visualised as box plots defining the distribution of expression levels across these genes in the samples. Genotypes are distinguished by color: wild-type samples are displayed in purple, *rad21*$^{-/-}$ in blue, *rad21*$^{+/-}$ in green, and *stag2b*$^{-/-}$ samples in yellow. For each panel, the dotted line indicates the level of expression in wild type.

Development • Supplementary information

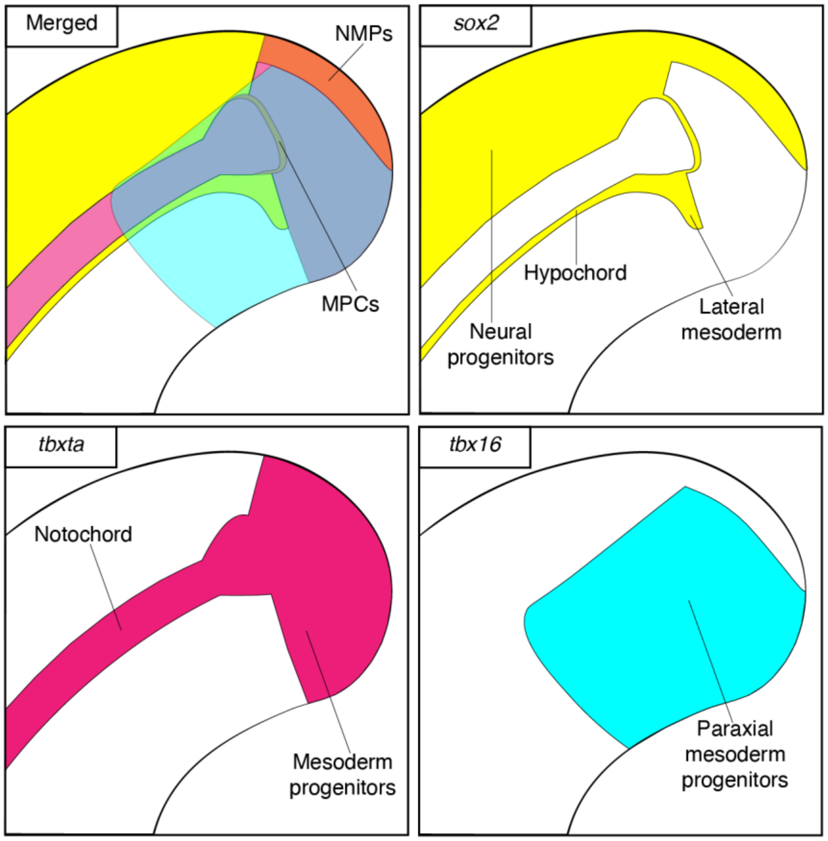

Fig. S7. Expression pattern of marker genes in the tailbud at the 16-somite stage.

Schematic depicts the regions in the tailbud and progenitor types where expression of *sox2* (yellow), *tbxta* (magenta) and *tbx16* (cyan) expression is expected.

Development • Supplementary information

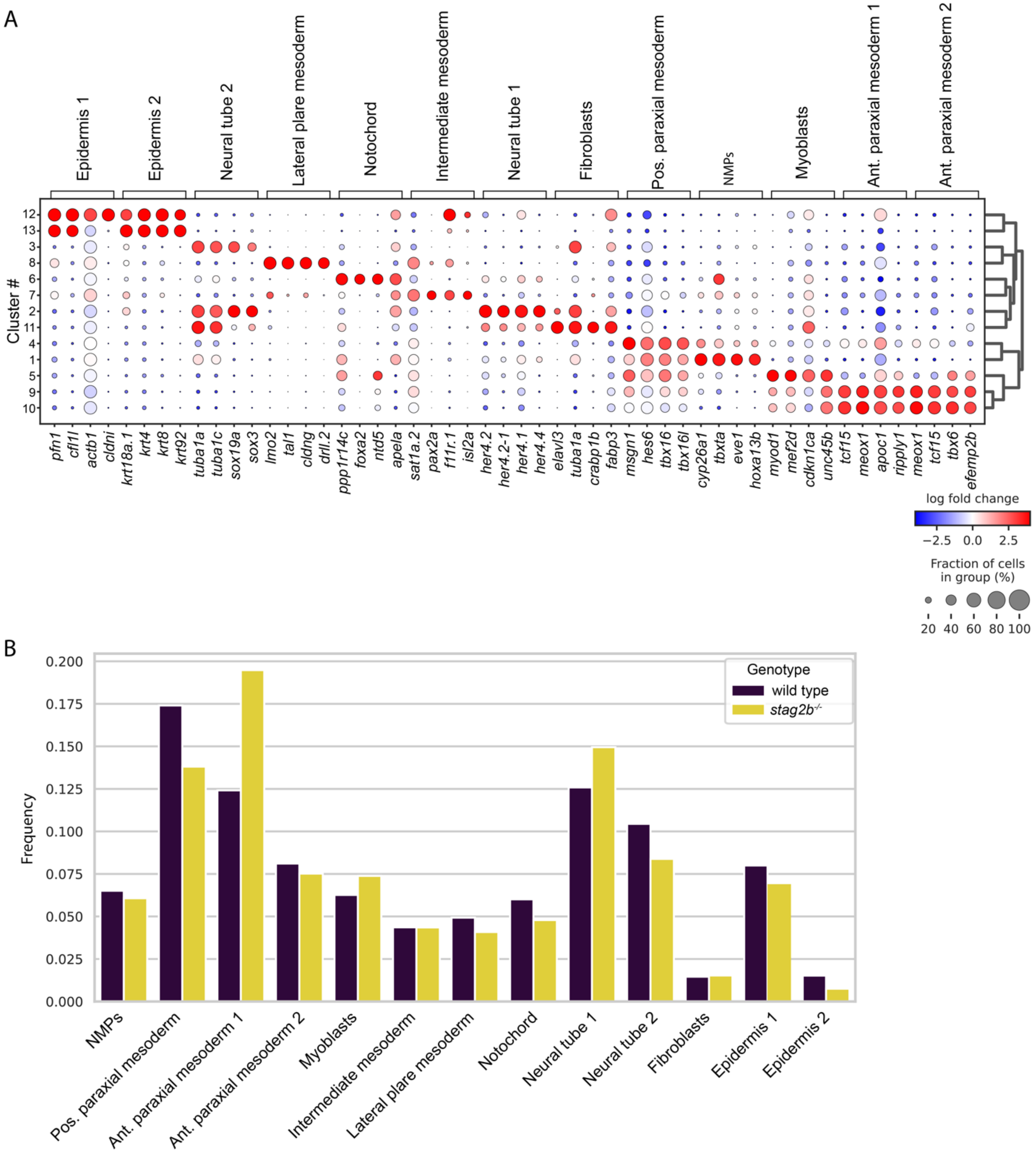

Fig. S8. Cell population analysis of single cell RNA sequencing of *stag2b*$^{-/-}$ mutant tailbuds compared with wild type.

(A) Dot plot depicting the expression of the top 4 marker genes per cell population cluster identified (see Fig. 7A). The dot size scales with the fraction of cells expressing the gene, and the dot color indicates the log fold change between the clusters. **(B)** Differences in the proportion of cell types between wild-type and *stag2b*$^{-/-}$ tailbuds among clusters as shown in Fig. 7A.

Development • Supplementary information

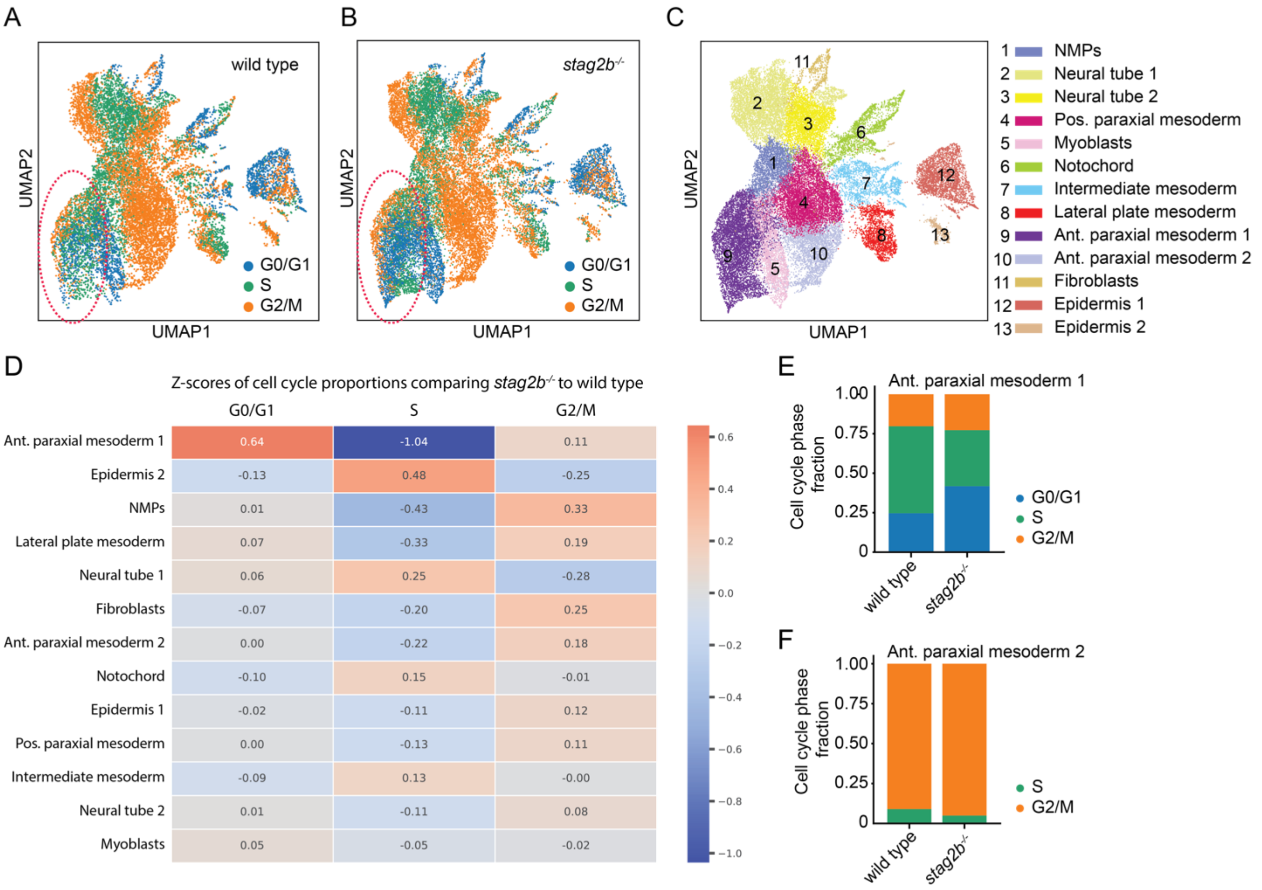

Fig. S9. Cell cycle phase analysis of single cell data from *stag2b*$^{-/-}$ and wild-type tailbuds. **(A-B)** UMAP of wild-type and *stag2b*$^{-/-}$ single cell data showing cell cycle phases in blue (G0/G1), green (S) and orange (G2/M). Dashed red outline indicates the anterior paraxial mesoderm cluster 1. All cluster annotations for the integrated dataset in **(C)**. **(D)** Ranked heatmap of z-scores of *stag2b*$^{-/-}$ cell clusters comparing cell cycle phase proportions. Red indicates an increase and blue a decrease in cell cycle phase compared to wild type. **(E)** Stacked bar plot of cell cycle phase fractions comparing anterior paraxial mesoderm 1 cluster in wild type with *stag2b*$^{-/-}$. **(F)** Stacked bar plot of cell cycle phase fractions comparing anterior paraxial mesoderm 2 cluster in wild type with *stag2b*$^{-/-}$.

Development • Supplementary information

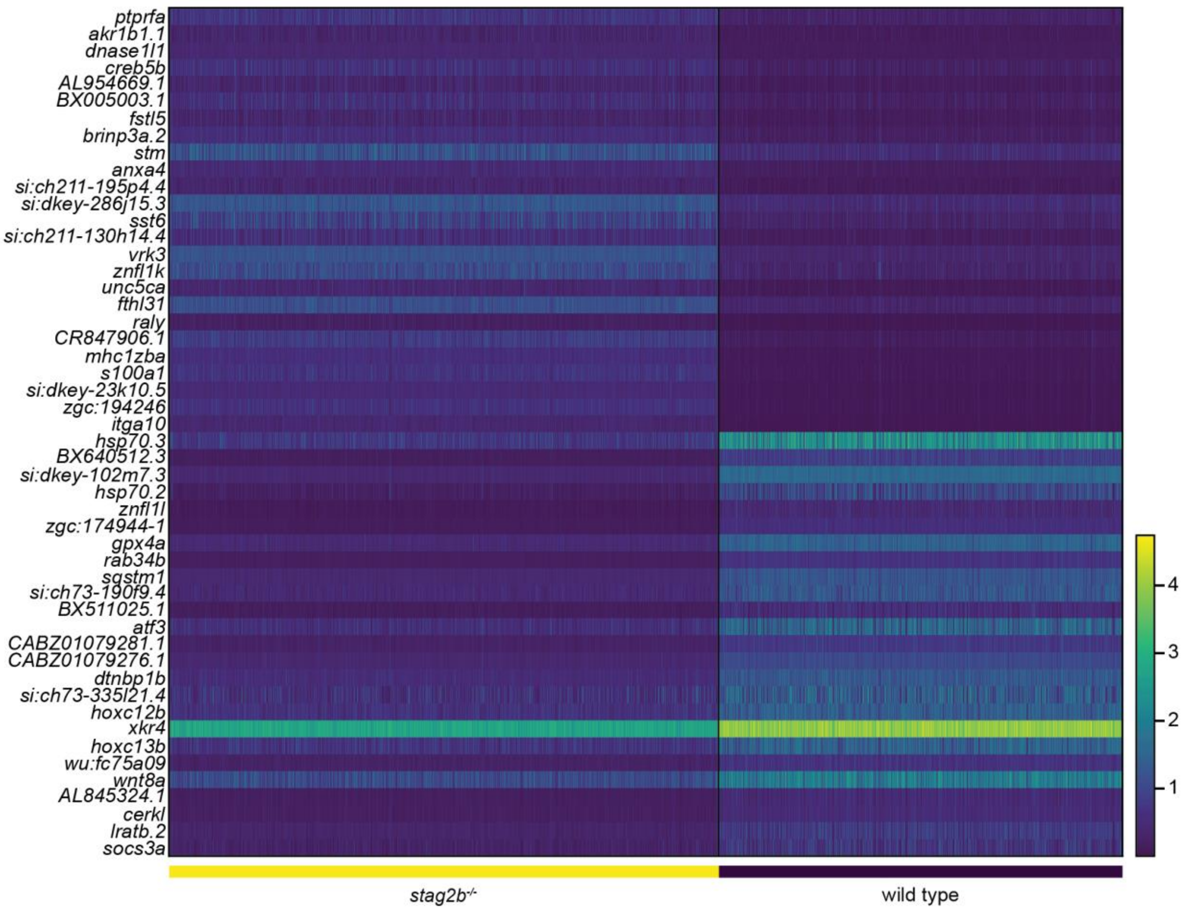

Fig. S10. Heat map showing the top 25 differentially up- and downregulated genes between wild-type and *stag2b*$^{-/-}$ NMP single cell RNA-seq data. For a full list see Table S4.

Development • Supplementary information

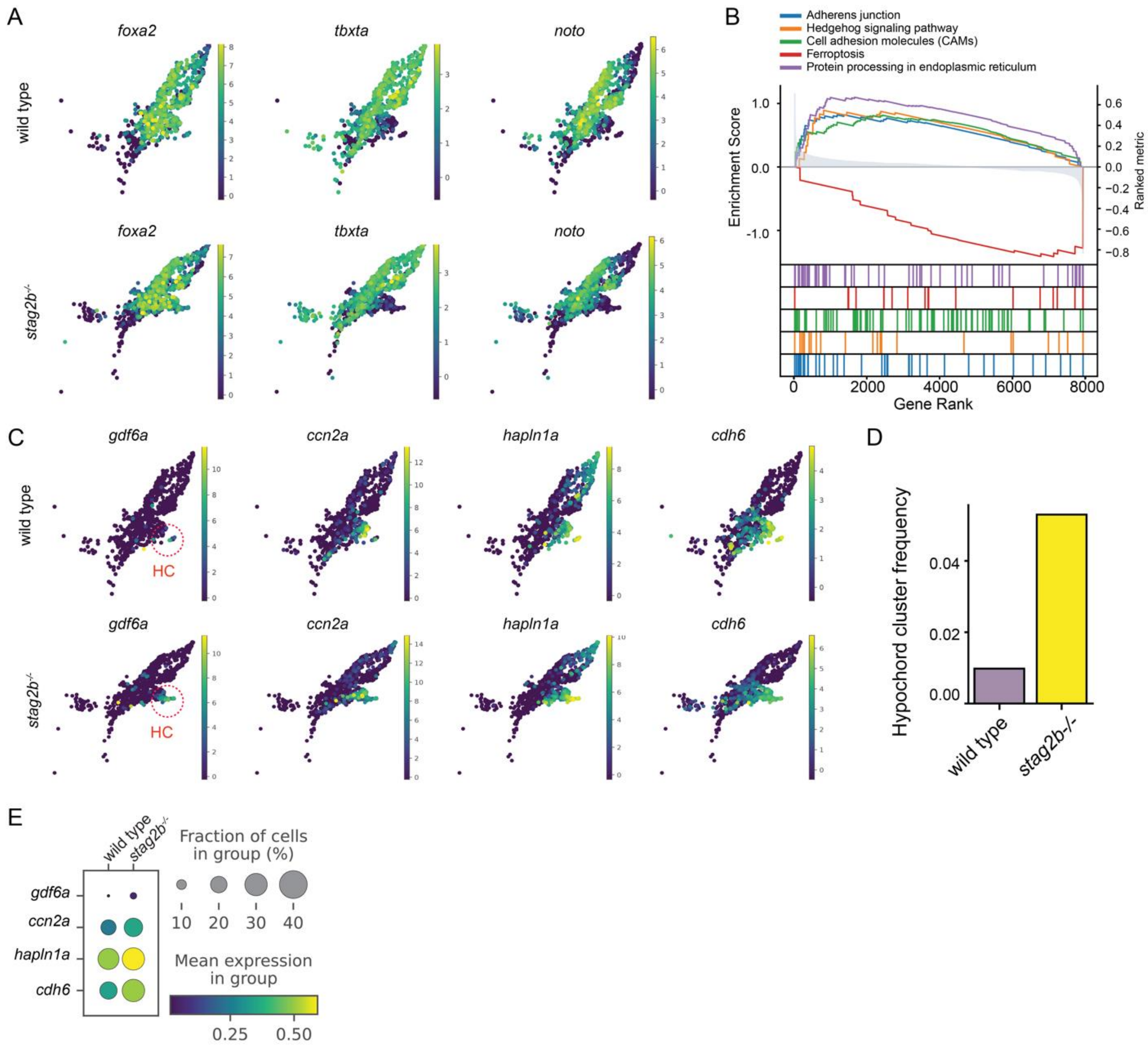

Fig. S11. Notochord single cell analysis shows upregulation of Hedgehog signaling with an expansion of hypochord cells in *stag2b*$^{-/-}$ tailbuds. **(A)** UMAP plot of notochord markers *fox2a, tbxta* and *noto* showing their expression in wild-type (top) and *stag2b*$^{-/-}$ (bottom) notochord cluster. **(B)**. Gene set enrichment analysis of differentially expressed genes derived from pseudo bulk analysis comparing the notochord cluster in wild-type and *stag2b*$^{-/-}$ tailbud single cell data. **(C)** UMAP of hypochord markers *gdf6a, ccn2a, hapln1a* and *cdh6* in wild-type (top) and *stag2b*$^{-/-}$ (bottom) notochord cluster. **(D)** Bar plot of hypochord-marked cells as proportion of cells in the notochord cluster in wild-type and *stag2b*$^{-/-}$ tailbud single data. **(E)** Dot plot of hypochord markers in the notochord cluster comparing wild-type and *stag2b*$^{-/-}$ tailbuds.

Development • Supplementary information

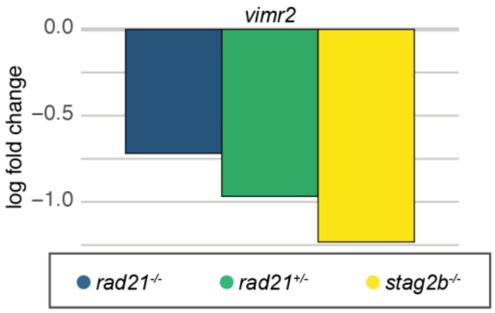

Fig. S12. *vimr2* expression in cohesin mutants. The bar graph displays the log2 fold change (5% FDR) for *vimr2* transcripts in *rad21*$^{-/-}$ (blue), *rad21*$^{+/-}$ (green) and *stag2b*$^{-/-}$ (yellow) tailbuds compared to wild-type tailbuds.

Development • Supplementary information

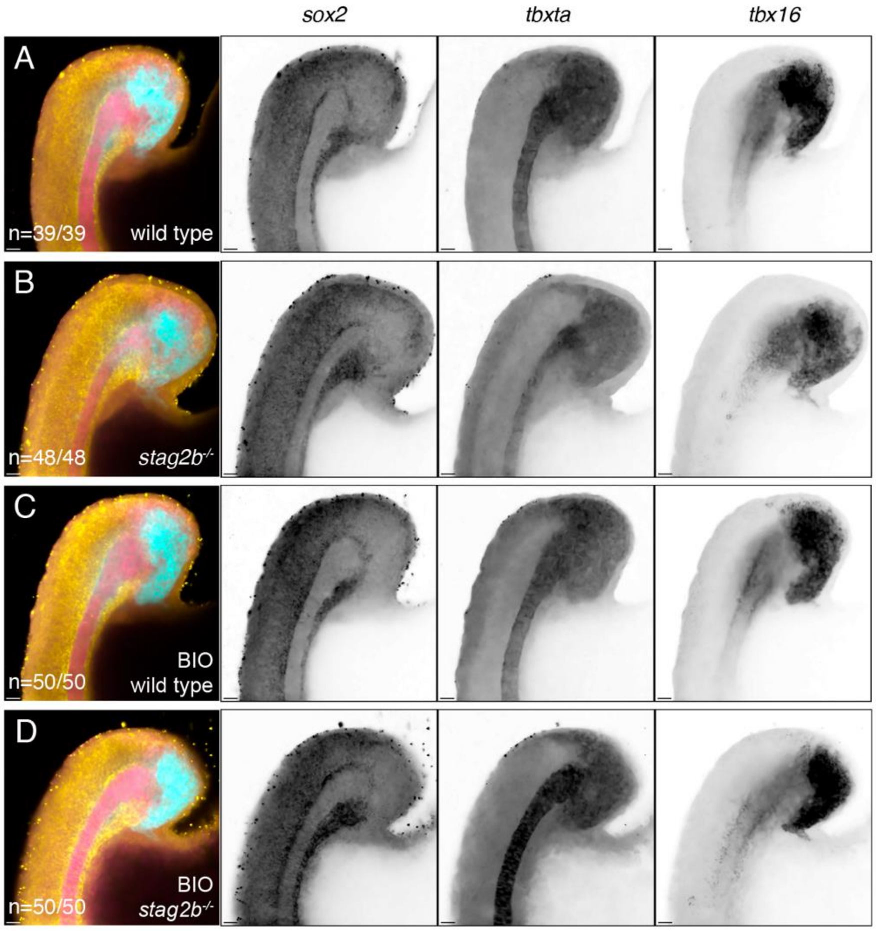

Fig. S13. Wnt stimulation rescues the notochord phenotype in *stag2b*$^{-/-}$ mutants.
(A-D) Expression patterns of *sox2*, tbxta, and *tbx16* in wild-type (A, C) and *stag2b*$^{-/-}$ (B, D) zebrafish tailbuds with (C, D) and without (A, B) Wnt stimulation. Images are maximum intensity projections of 3 (4.8 µm) optical sections. Scale bars are 20 µm. The number of embryos with each expression pattern out of the total analyzed is noted at bottom left of the left-hand panels.

Development • Supplementary information

Table S1. Cell cycle genes (Reactome PA) significantly (5% FDR) dysregulated in homozygous mutant *rad21* tailbuds.

Available for download at
https://journals.biologists.com/dev/article-lookup/doi/10.1242/dev.202593#supplementary-data

Table S2. Cell cycle genes (Reactome PA) significantly (5% FDR) dysregulated in heterozygous mutant *rad21* tailbuds.

Available for download at
https://journals.biologists.com/dev/article-lookup/doi/10.1242/dev.202593#supplementary-data

Table S3. Cell cycle genes (Reactome PA) significantly (5% FDR) dysregulated in homozygous mutant *stag2b* tailbuds.

Available for download at
https://journals.biologists.com/dev/article-lookup/doi/10.1242/dev.202593#supplementary-data

Table S4. Significantly (5% FDR) differentially expressed genes in the NMP subset comparing homozygous *stag2b*$^{-/-}$ and wild-type tailbuds, related to Figure 7C,E and Fig. S10.

Available for download at
https://journals.biologists.com/dev/article-lookup/doi/10.1242/dev.202593#supplementary-data

Table S5. Gene set enrichment analysis results comparing NMPs of homozygous *stag2b*$^{-/-}$ and wild-type tailbuds, related to Fig. 7C.

Available for download at
https://journals.biologists.com/dev/article-lookup/doi/10.1242/dev.202593#supplementary-data

Table S6. Gene list related to canonical Wnt signalling score, related to Fig. 7D.

Available for download at
https://journals.biologists.com/dev/article-lookup/doi/10.1242/dev.202593#supplementary-data

Table S7. Wilcoxon rank sum test statistics for each cell cluster comparing the Wnt signaling score shown in Fig. 7D among homozygous *stag2b*$^{-/-}$ and wild-type genotypes.

Available for download at
https://journals.biologists.com/dev/article-lookup/doi/10.1242/dev.202593#supplementary-data

Table S8. Significantly (5% FDR) differentially expressed genes in the notochord subset comparing homozygous *stag2b*$^{-/-}$ and wild-type tailbuds, related to Fig. S11.

Available for download at
https://journals.biologists.com/dev/article-lookup/doi/10.1242/dev.202593#supplementary-data

Table S9. Gene set enrichment analysis results comparing the notochord cluster of homozygous *stag2b*$^{-/-}$ and wild-type tailbuds, related to Fig. S11.

Available for download at
https://journals.biologists.com/dev/article-lookup/doi/10.1242/dev.202593#supplementary-data

Development • Supplementary information
