# Supplementary material for: Cohesin composition and dosage independently affect early development in zebrafish
Source: Development. 2024 Aug 1;151(15):dev202593. doi: 10.1242/dev.202593 (PMC11317101; doi:10.1242/dev.202593)
Supplement: Supplementary file 1 [file develop-151-202593_review_history.pdf]

Cohesin composition and dosage independently affect early development in zebrafish

Anastasia A Labudina, Michael Meier, Gregory Gimenez, David Tatarakis, Sarada Ketharnathan, Bridget Mackie, Thomas F Schilling, Jisha Antony and Julia A Horsfield
DOI: 10.1242/dev.202593

Editor: Steve Wilson

Review timeline

Original submission:	7 December 2023
Editorial decision:	30 January 2024
First revision received:	28 April 2024
Editorial decision:	18 June 2024
Second revision received:	21 June 2024
Accepted:	27 June 2024

Original submission

First decision letter

MS ID#: DEVELOP/2023/202593

MS TITLE: Cohesin composition and dosage independently affect early development in zebrafish

AUTHORS: Anastasia A Labudina, Michael Meier, Gregory Gimenez, David Tatarakis, Sarada Ketharnathan, Bridget Mackie, Thomas F Schilling, Jisha Antony, and Julia A Horsfield

I have now received all the referees' reports on the above manuscript, and have reached a decision. The referees' comments are appended below, or you can access them online: please go to BenchPress and click on the 'Manuscripts with Decisions' queue in the Author Area.

As you will see, the referees express considerable interest in your work, but have some significant criticisms and suggestions to improve the data presented in your manuscript and how the data informs how Cohesin might be functioning in the processes you study. If you are able to revise the manuscript along the lines suggested, I will be happy receive a revised version of the manuscript. Please also note that Development will normally permit only one round of major revision. If it would be helpful, you are welcome to contact us to discuss your revision in greater detail. Please send us a point-by-point response indicating your plans for addressing the referees’ comments, and we will look over this and provide further guidance.

Please attend to all of the reviewers' comments and ensure that you clearly highlight all changes made in the revised manuscript. Please avoid using 'Tracked changes' in Word files as these are lost in PDF conversion. I should be grateful if you would also provide a point-by-point response detailing how you have dealt with the points raised by the reviewers in the 'Response to Reviewers' box. If you do not agree with any of their criticisms or suggestions please explain clearly why this is so.

Reviewer 1

Advance summary and potential significance to field

© 2024. Published by The Company of Biologists under the terms of the Creative Commons Attribution License (https://creativecommons.org/licenses/by/4.0/).

The manuscript describes the different transcriptional and developmental outcomes resulting from reducing the overall amount of cohesin compared to altering its composition. To investigate this, the authors studied the consequences of depleting Rad21 or Stag2 in zebrafish embryos, using the embryonic tailbud as a stem cell model. Both rad21 and stag2 mutants exhibit deficiencies in mesoderm induction, but these arise from different molecular mechanisms. Given that human cohesinopathies are characterized by germline mutations in cohesin subunits or its regulators, these observations have potential implications for understanding and managing cohesinopathies.

Comments for the author

Minor comments
1. In Figure 1, letter D is missing in the corresponding panel.
2. In Figure 5, fluorescent images of the HCR RNA-FISH are difficult to see. Would it be possible to make the images bigger? It would help to see the phenotypes.
3. In Figure 7B, I am not sure if the scale numbers are the correct, as the assigned colors in the legend do not correspond to the ones in the figure.
4. The names of the Supplementary Figures from Figure S3 onwards are not properly annotated (Figure S4 is written as Figure S1, Figure S5 title is written as Figure S2…).
5. In the legend of Figure S8 according to the text (but named as Figure S5 in the legend), there is a reference to Supplementary Data S1, but it should be written Data S4.
6. In line 171, stag1b-/-; stag2b-/- embryos at 48 hpf correspond to (Fig. 1H,J) instead of (Fig. 1G-J).
7. You show in Figure S2 the normalized transcript counts of various cohesin subunits in the different genotypes. Do the observed transcriptional changes correlate with alterations at the protein level, affecting the assembly of the complex. Have you explored anything in this regard?

Reviewer 2

Advance summary and potential significance to field

Cohesin, a multi-protein complex, plays critical roles in cell proliferation and gene expression. A significant amount of effort has been put into understanding the molecular basis underlying the regulation of chromatin structure by this complex. However, much is known about the complicated relationship between the subunits of the complex, especially in a developmental context. In this manuscript, Labudina and colleagues aim to understand the developmental consequences when different cohesion subunits are mutated.
The authors analyzed the morphological and molecular phenotype of rad21-/-, rad21+/-, stag2b-/- and stag1b-/-;stag2b-/- and found that mutations of stag2b and rad21 have different developmental consequences. They discovered that the thickness of notochord is significantly narrower in stag2b mutants, and further revealed that Wnt signaling is likely underlying this narrow notochord phenotype. Interestingly, they found that stag2b mutation also rescues the narrow notochord phenotype.
Although this study does not provide molecular mechanisms explaining why mutations in different cohesion subunits lead to different developmental consequences, many findings are still informative to the developmental and stem cell community. However, many aspects need to be addressed before publication.

Comments for the author

Major:
1. The first subtitle in the result section, “Stag1b and Stag2b are the main functional Stags in zebrafish”, is not well-supported. The absence of a stag2a mutant line limits the ability to determine whether Stag2a is functionally dispensable. Even stag2a single mutant is viable, without exerting all combinations of double and triple mutants among Stag1a, Stag1b, Stag2a, and Stag2b, one cannot conclude that “Stag1b and Stag2b are the main functional Stags in zebrafish”. I suggest the authors change this subtitle.
2. While the differentiation between the function of cohesin complex quantity versus composition is conceptually interesting, the current results in the manuscript are insufficient to

© 2024. Published by The Company of Biologists under the terms of the Creative Commons Attribution License (https://creativecommons.org/licenses/by/4.0/).

address this distinction. Without the support of biochemistry and genomics data, it is only an assumption that stag2b mutants represent cohesin composition. For example, if cohesin composition is behind the narrow notochord phenotype in the stag2b mutants, how do we explain the rescue of this phenotype by mutation of stag1b?
3. Genetic backgrounds need to be taken into consideration when comparing transcriptomes of different fish lines. Assuming rad21-/- and rad21+/- embryos are siblings, they will share more similarities in genetic backgrounds; this may be one of the reasons for the higher similarity between rad21-/- and rad21+/- embryos. Thus, when comparing wildtype embryos, rad21 mutants, and the stag2b-/- mutant, which differentially expressed genes are due to genetic backgrounds versus cohesin function? More importantly, if the differentially expressed genes between rad21+/- and wildtype embryos are due to cohesin dosage effects, can one rescue the expression of these differentially expressed genes by overexpression of a specific dosage of rad21 in the rad21+/- background?
4. Two concerns regarding Figure 1G-J: a. The cell density and shape in rad21-/- and stag1b-/-;stag2b-/- mutants are dramatically different; how can we ensure the fairness of this comparison? In other words, how can we ensure that the differences observed are not due to cell type differences? b. Please specify how many embryos are imaged and quantify the observed phenotype (What's the percentage in different embryos? Are the differences significant?).
5. The sc-RNA seq data may provide insights into the molecular basis underlying the narrow notochord in the stag2b mutant and the rescue of this phenotype by Wnt signaling and stag1b mutation. What are the genes that are differentially expressed between the stag2b and wildtype embryos in the notochord?
Minor:
1. Please correct the supplementary figure number: currently Figure S1-S3, Figure S1-S7 (should be Figure S4-S10)
2. Line 191: Flow cytometry showed no significant differences in the proportions of cells. What is the statistical analysis used here?
3. Citing the references when mentioning the analysis used will facilitate the audience, even though these references were cited in the method section. Some examples:
Line 224-225: Differential gene expression analysis (citation)
Line 232: Metascape (citation)
Line 239: Reactome (citation)
Line 349: interaction analysis (citation)
4. Figure 5: please use a different color to outline the increased sox2 expression in the NMP region. The current color is challenging to discern.
5. Missing statistics: Figure 8D, Figure S2, the second Figure S2 (should be figure S5)
6. Please add more details about the method used to facilitate the readers. For example, for the RNA-seq data, please specify which Ensembl release was used and what software was used to plot different plots.
7. Line 561, please cite Ensembl.

Reviewer 3

Advance summary and potential significance to field

Labudina et al. present their manuscript entitled "Cohesin composition and dosage independently affect early development in zebrafish" in which they investigate developmental and genomic impacts of Cohesin disruption during zebrafish development. They focus on Rad21, the primary subunit of the Cohesin complex, and Stag2, an ancillary subunit which has been shown previously to have Cohesin independent function. They begin with general phenotypic characterization, showing that combined loss of Stag1 and Stag2 phenocopies Rad21 loss, and defects individual mutant do not seem to be the result of impaired cell division. They next investigate developmental defects resulting from Rad21 and Stag2 deficiency finding that rad21 deficiency leads to a block in NMP differentiation, while stag2 deficiency results in a narrow notochord. This leads to authors to investigate cell type specification defects, and they utilize single-cell RNA-Seq for these purposes. Here they find that paraxial mesoderm is most affected, showing evidence for fewer cells in stag2 mutant embryos, as well as dysregulation of gene expression. The authors speculate based on impacted genes that failed Wnt signaling might underlie these defects, and demonstrate this to be the case by treating embryos with BIO - a Wnt agonist. This result implies that sub-unit specific

© 2024. Published by The Company of Biologists under the terms of the Creative Commons Attribution License (https://creativecommons.org/licenses/by/4.0/).

forms of the Cohesin complex impact Wnt signaling. If correct, this would be an interesting outcome and a novel mechanism in development.

Comments for the author

Labudina et al. present their manuscript entitled "Cohesin composition and dosage independently affect early development in zebrafish" in which they investigate developmental and genomic impacts of Cohesin disruption during zebrafish development. They focus on Rad21, the primary subunit of the Cohesin complex, and Stag2, an ancillary subunit which has been shown previously to have Cohesin independent function. They begin with general phenotypic characterization, showing that combined loss of Stag1 and Stag2 phenocopies Rad21 loss, and defects individual mutant do not seem to be the result of impaired cell division. They next investigate developmental defects resulting from Rad21 and Stag2 deficiency finding that rad21 deficiency leads to a block in NMP differentiation, while stag2 deficiency results in a narrow notochord. This leads to authors to investigate cell type specification defects, and they utilize single-cell RNA-Seq for these purposes. Here they find that paraxial mesoderm is most affected, showing evidence for fewer cells in stag2 mutant embryos, as well as dysregulation of gene expression. The authors speculate based on impacted genes that failed Wnt signaling might underlie these defects, and demonstrate this to be the case by treating embryos with BIO - a Wnt agonist. This result implies that sub-unit specific forms of the Cohesin complex impact Wnt signaling. If correct, this would be an interesting outcome and a novel mechanism in development.

This is an interesting study which provides novel insight into nuanced aspects of Cohesion function during development, but I do think that additional experimentation is necessary to provide sufficient mechanistic details.

1) Overall, I find the experiments to be quite interesting, but there is a general lack of interpretation. I found their conclusions to be wholly unsatisfying and urge the authors to speculate more about what mechanisms might underlie their experimental outcomes. Even the conclusion paragraphs at the end of the results section and throughout the discussion section, the authors are simply re-hashing their results. The text is almost completely descriptive, focused specifically on their experimental outcomes, and there is a lack of broad biological conclusions relating to these outcomes. It is therefore left to the reader to piece together what all of their conclusions might mean. For example what does it mean that a specific subunit of Cohesin, namely Stag2, specifically impacts Wnt signaling? Why doesn't Stag1 or Rad21 loss lead to the same outcomes?
Do these specific subunits allow Cohesin to take on unique or locus-specific functions, as implied but not directly stated? Even the title and abstract dont sufficiently capture the boarder impacts of the study.

2) The authors imply that Stag2, and perhaps Stag1, might have function independent of Rad21, and they imply in the introduction and discussion that enhancer regulation (and/or CTCF) might be involved, but the authors do not investigate this hypothesis at all. I suspect based on the results that Stag2 might be particularly important for enhancers that either respond to or stimulate Wnt signaling. What are these enhancers? Could the authors use ATAC-Seq or ChIP-Seq in the tailbud to identify enhancer defects, those that are disrupted from Stag2 loss of function?

3) I found their gene expression analysis to be insufficient. For example authors state on line 46 that transcriptional changes in rad21 mutants resemble stag2b mutant, and they refer to a PCA plot in Figure 3B. This PCA plot does not provide any comparison of differentially expressed genes. The authors do assess gene expression changes in 3C-E, but they never compare between these groups, as suggested by the text. Such a comparison would be very valuable for assessing outcomes of the mutations. Are differentially expressed genes in one genotype also differentially expressed in another? Do these genes change expression in a similar manner? A thorough gene-by-gene analysis is necessary for this type of comparison rather than a rudimentary PCA.

4) In Figure 7B authors use somewhat uncommon and low-resolution method for comparing gene expression patterns between single-cell RNA-Seq datasets. They argue that changes in gene expression are most significant for cells within clusters 9 and 10. It would be much more convincing

© 2024. Published by The Company of Biologists under the terms of the Creative Commons Attribution License (https://creativecommons.org/licenses/by/4.0/).

if the authors sub-sampled populations of cells within each cluster and then applied pseudo-bulk analysis within each sub-population to establish gene expression profiles (simulating replicates), which would then allow them to performed traditional DESeq2 and identify differentially expressed genes. Not only would this provide an orthogonal means to assess gene expression differences, but it would also allow the researchers to identify the most differentially expressed genes, and then assess gene ontology or pathway analysis.

5) Authors rely pretty heavily on a single method for manipulating wnt signaling. The paper would be much stronger if there were orthogonal strategies to disrupt wnt signaling. I could imagine inhibiting wnt or using a morpholino to inhibit expression of wnt8. The expectation is that these inhibition experiments would phenocopy Stag2 mutants, or perhaps the stag2 deficient embryos would be more sensitive to wnt inhibition.

First revision

Author response to reviewers' comments

Please find below our point-by-point responses to the reviewers' comments.

Reviewer 1 Comments for the Author:

Minor comments

1. In Figure 1, letter D is missing in the corresponding panel.

Corrected, thank you.

2. In Figure 5, fluorescent images of the HCR RNA-FISH are difficult to see. Would it be possible to make the images bigger? It would help to see the phenotypes.

Thank you for the comments, we revised the HCR figures to make them easier to see. We now provide a single panel of the merge and separate black-and-white images of each individual expression pattern for *sox2*, *tbxta* and *tbx16*. **Please see revised Figure 5 and revised Figure S13.**

3. In Figure 7B, I am not sure if the scale numbers are the correct, as the assigned colors in the legend do not correspond to the ones in the figure.

We agree that the legend to this figure was confusingly positioned so that the legend appeared to apply to left and right panels when it only applied to the left panel. However, we have added substantial extra analyses of the scRNA-seq data and this led to a **substantially revised Figure 7** and **two new supplementary figures, S9 and S11**. We believe that this has improved the clarity and interpretation of the scRNA-seq data.

4. The names of the Supplementary Figures from Figure S3 onwards are not properly annotated (Figure S4 is written as Figure S1, Figure S5 title is written as Figure S2...).

Corrected, thank you for spotting this error.

5. In the legend of Figure S8 according to the text (but named as Figure S5 in the legend), there is a reference to Supplementary Data S1, but it should be written Data S4.

Corrected, thank you for spotting this error. Please note that we have added extra data tables to support the manuscript so the ordering might be different.

6. In line 171, stag1b-/-; stag2b-/- embryos at 48 hpf correspond to (Fig. 1H,J) instead of (Fig. 1G-J).

© 2024. Published by The Company of Biologists under the terms of the Creative Commons Attribution License (https://creativecommons.org/licenses/by/4.0/).

Corrected, thank you for spotting this error.

7. You show in Figure S2 the normalized transcript counts of various cohesin subunits in the different genotypes. Do the observed transcriptional changes correlate with alterations at the protein level, affecting the assembly of the complex. Have you explored anything in this regard?

In Horsfield et al 2007 (PMID: 17567667, Fig 3), and Rhodes et al 2010 (PMC2941799, Fig S4), we measured protein levels of Rad21 in wild type, sibs and mutants and our previous results show that protein levels reflect transcript levels. Meier et al 2018 (PMID: 29158440) and Schuster et al 2015 (PMC4654055) also show dosing of Rad21 protein that scales with morpholino targeting of *rad21* transcript. Unfortunately we don't have a Stag2 antibody that works in zebrafish, but please see our response to reviewer 2 outlining our reasoning for the maintenance of cohesin levels in these homozygous viable zebrafish.

Reviewer 2 Comments for the Author:

Major:
1. The first subtitle in the result section, "Stag1b and Stag2b are the main functional Stags in zebrafish", is not well-supported. The absence of a stag2a mutant line limits the ability to determine whether Stag2a is functionally dispensable. Even stag2a single mutant is viable, without exerting all combinations of double and triple mutants among Stag1a, Stag1b, Stag2a, and Stag2b, one cannot conclude that "Stag1b and Stag2b are the main functional Stags in zebrafish". I suggest the authors change this subtitle.

We agree with the reviewer that it is somewhat unsatisfactory not to have a *stag2a* mutant so that all genetic combinations of stag genes could be explored. However, we remain convinced that it is accurate to state that Stag1b and Stag2b are the main functional stags in zebrafish. Evidence for this is as follows:

1. Homozygous loss of zebrafish Stag1b and Stag2b is lethal in combination (Fig 1), as previously observed for combined loss of human STAG1 and STAG2 (van der Lelij 2020 PMC7266993; 2017 PMC5531830).
2. Loss of zebrafish Stag1b and Stag2b phenocopies the null cohesin mutation *rad21* (Fig 1), meaning that remaining Stags1a and 2a cannot rescue progression through the cell cycle, or *runx1* expression - i.e., only Stag1b and Stag2b can function in this respect. This implies that Stag2a is functionally dispensable for cohesin, because it's not able to rescue loss of Stag2b.
3. In contrast homozygous loss of both zebrafish Stag1a and Stag2b is double homozygous viable with a minor 5% penetrant haematopoietic defect (Fig S1), meaning that only Stag1b of the two Stag1s is indispensable.
4. *stag2a* is the least expressed of all the *stag* genes during zebrafish development (Fig S3, and Ketharnathan et al 2020, please see the figure below from that study) which is consistent with it not having a substantial function especially during the tailbud stages analysed in the present manuscript. This contrasts with the high expression of Stag1b and 2b. We have added a supporting line to the manuscript (line 200) to emphasize the absence of stag2a expression at tailbud stages.

NOTE: Figure provided for reviewer has been removed. It showed Figure 1 from **Ketharnathan, S., Labudina, A. and Horsfield, J. A.** (2020), Cohesin components Stag1 and Stag2 differentially influence haematopoietic mesoderm development in zebrafish embryos. Front. Cell Dev. Biol. 8, 617545. doi:10.3389/fcell.2020.617545. We have removed the unpublished data that had been provided for the referees in confidence.

2. While the differentiation between the function of cohesin complex quantity versus composition is conceptually interesting, the current results in the manuscript are insufficient to address this distinction. Without the support of biochemistry and genomics data, it is only an assumption that stag2b mutants represent cohesin composition.

We agree that transcription data is only one part of a whole picture, but we remain convinced that the *stag2b* and *rad21* mutants reflect composition and dosage respectively, supported by the data presented in our manuscript and by previous work.

© 2024. Published by The Company of Biologists under the terms of the Creative Commons Attribution License (https://creativecommons.org/licenses/by/4.0/).

- If homozygous mutations in *stag2b* and *stag1b* are lethal in combination, then in the absence of Stag2b, Stag1b must be compensating. Because *stag2b* mutants are homozygous viable, fertile, and maintain expression of all other cohesin subunits (Fig S3), we deduce that there must be a normal amount of cohesin present that predominantly contains Stag1b = altered composition of cohesin.
- In contrast, in this manuscript (Fig S3) and in our previous work (Horsfield et al 2007, PMID: 17567667; Schuster et al 2015, PMC4654055) we showed that genetically halving the dose of (or using a morpholino targeting) *rad21* reduces its transcript, which elicits a partial l.o.f. phenotype. In the present manuscript we confirm that gene expression changes and phenotype scale with *rad21* dose (Fig 3C,D; Fig 5B,C) and this demonstrates that *rad21* transcript likely limits the amount of complexes that can form.
- Other studies support this interpretation. Halving the dose of *Rad21* also leads to a phenotype in mice (Xu et al 2010 PLOS ONE 5(8): e12112). Another study cited in our paper shows that Rad21 quantitatively promotes the loading of complexes onto DNA (Sun et al., 2023), implying that modulating dose of *rad21* is proportional to the number of cohesin complexes on DNA.

For example, if cohesin composition is behind the narrow notochord phenotype in the stag2b mutants, how do we explain the rescue of this phenotype by mutation of stag1b?

As explained above, additional mutation of *stag1b* gives rise to the *stag1b/stag2b* double mutant, which has no functional cohesin - i.e., it phenocopies the *rad21* null mutant, because no other Stags can compensate. Therefore the *stag1b/stag2b* double mutant is not composition-changing but rather cohesin loss-of-function. To clarify, mention of “rescue” by *stag1b* mutation has been removed from the last section of the introduction and in the results - instead, the idea that complete cohesin loss is likely epistatic to the effects of *stag2b* mutation on the notochord is emphasised.

3. Genetic backgrounds need to be taken into consideration when comparing transcriptomes of different fish lines. Assuming rad21-/- and rad21+/- embryos are siblings, they will share more similarities in genetic backgrounds; this may be one of the reasons for the higher similarity between rad21-/- and rad21+/- embryos. Thus, when comparing wildtype embryos, rad21 mutants, and the stag2b-/- mutant, which differentially expressed genes are due to genetic backgrounds versus cohesin function?

In our study, the wild type strain WIK served as a consistent genetic background across all cohesin deficient zebrafish lines. Importantly, the comparison between wildtype (WT) embryos and mutants is based on DESeq analysis, where WT embryos are not genetically related to any of the mutant samples and each comparison is made with WT (WT vs mut).

We agree that *rad21+/-* and *rad21-/-* are more closely related and that the shared genetic background may contribute to the outcomes of the bioinformatic analysis. However, some downstream gene expression changes for *rad21+/-* were shown to be at an intermediate level between *rad21-/-* and wild type. Evidence in the present manuscript can be found in (for e.g.) Fig S6, *tbx16* and *msgn1*; see also Horsfield et al 2007 (PMID: 17567667), Rhodes et al 2010, (PMC2941799). Please find below an excerpt from Fig 2 of Rhodes et al below, showing downstream gene regulation in embryos from an incross of *rad21+/-*, in which all individuals are related This evidence supports a dosage effect for *rad21*, and that the relationship between *ad21+/-* and *rad21-/-* is not accounted for by their shared background.

NOTE: Figure provided for reviewer has been removed. It showed Figure 2 from **Rhodes, J. M., Bentley, F. K., Print, C. G., Dorsett, D., Misulovin, Z., Dickinson, E. J., Crosier, K. E., Crosier, P. S. and Horsfield, J. A.** (2010). Positive regulation of c-Myc by cohesin is direct, and evolutionarily conserved. Dev. Biol. 344, 637-649. doi:10.1016/j.ydbio.2010.05.493. We have removed unpublished data that had been provided for the referees in confidence.

More importantly, if the differentially expressed genes between rad21+/- and wildtype embryos are due to cohesin dosage effects, can one rescue the expression of these differentially

© 2024. Published by The Company of Biologists under the terms of the Creative Commons Attribution License (https://creativecommons.org/licenses/by/4.0/).

expressed genes by overexpression of a specific dosage of rad21 in the rad21+/- background?

The zebrafish *rad21* transcript will rescue *rad21-/-* mutants, as we showed in Horsfield et al 2007 (PMID: 17567667) and Deardorff et al 2012 (PMID: 22633399). It's difficult to overexpress the *rad21* transcript without some toxicity, which would be problematic for a bulk RNA-seq experiment that would need to contain hundreds of individuals injected with *rad21* transcript. We tried to perform a rescue experiment of *rad21+/-* dosage before, but we found that practically it is very difficult to overexpress *rad21* transcript with that kind of precision - instead in a previous study (Schuster et al 2015, PMC4654055) we used a tiny dose of a *rad21*-targeting morpholino injected into a *rad21+/-* incross, such that there was no phenotype with wild type embryos but the morpholino produced a phenotype in presumptive *rad21+/-* hets. In summary, our many published studies on the *rad21* mutant since 2007 provide considerable previous evidence for a dose effect, and we're confident that our previous and current data support the conclusions of the present manuscript.

4. Two concerns regarding Figure 1G-J: a. The cell density and shape in rad21-/- and stag1b-/-;stag2b-/- mutants are dramatically different; how can we ensure the fairness of this comparison? In other words, how can we ensure that the differences observed are not due to cell type differences?

The comparison in Figure 1 is not between *rad21-/-* and *stag1b-/-;stag2b-/-* mutants, it's between wild type and *stag1b-/-;stag2b-/-* mutants. G, I are wild type and H, J are the *stag1b/stag2b* double mutant, both at 48 hpf. G, I show normal mitoses and H, J show mitotic catastrophe in embryos that have lost all cohesin. Eventually every cell division is affected. We previously showed the same phenotype for *rad21* mutants (Horsfield et al 2007, PMID: 17567667).

b. Please specify how many embryos are imaged and quantify the observed phenotype (What's the percentage in different embryos? Are the differences significant?).

We didn't quantify these results because in *stag1b/stag2b* double mutants at the later stage of 48 hpf, every dividing cell eventually crashes in mitosis owing to lack of cohesin, which is what leads to the lethality of this mutant combination.

5. The sc-RNA seq data may provide insights into the molecular basis underlying the narrow notochord in the stag2b mutant and the rescue of this phenotype by Wnt signaling and stag1b mutation. What are the genes that are differentially expressed between the stag2b and wildtype embryos in the notochord?

This is a great question that prompted substantial further analyses of the single cell RNA-seq data. Our further analyses on clustered notochord cells showed increased activation of Hedgehog signalling in *stag2b-/-*. The data also suggest that increased Notch signalling from the higher number of paraxial mesoderm cells may increase in hypochord at the expense of notochord. The new information is presented in **revised Figure 7F, new Figure S11** and supported by **new Data S8, S9,** with corresponding changes in the main text starting at line 340 with the results for notochord explained starting line 373.

Minor:
1. Please correct the supplementary figure number: currently Figure S1-S3, Figure S1-S7 (should be Figure S4-S10)

Figure numbering has been corrected, thank you.

2. Line 191: Flow cytometry showed no significant differences in the proportions of cells. What is the statistical analysis used here?

This comment prompted us to examine the flow cytometry data more closely to answer the question, and we provide this information in a **new Figure S2**. We re-analysed the flow cytometry data using a tighter gate leaving out debris from the *rad21* samples, which resulted in fewer singlets per total events, see **new Figure S2A-D**. Using Cytoflow we quantified the percentage of cells in each phase and used a Mann-Whitney U test to test for significance ($P < 0.05$). We found

© 2024. Published by The Company of Biologists under the terms of the Creative Commons Attribution License (https://creativecommons.org/licenses/by/4.0/).

rad21-/- homozygous mutants to have significantly reduced S phase with no significant changes in the other genotypes, **new Figure S2F**.

3. Citing the references when mentioning the analysis used will facilitate the audience, even though these references were cited in the method section. Some examples:
Line 224-225: Differential gene expression analysis (citation)
Line 232: Metascape (citation)
Line 239: Reactome (citation)
Line 349: interaction analysis (citation)
Citations included as suggested.
4. Figure 5: please use a different color to outline the increased sox2 expression in the NMP region. The current color is challenging to discern.

Figure 5 has been revised to make the changes in expression of the tailbud markers clearer. We include higher magnification black and white images of the individual gene expression patterns, with just one colour panel showing the merge. **See Revised Figure 5.** We **revised Figure S13** similarly to achieve the same clarity.

5. Missing statistics: Figure 8D, Figure S2, the second Figure S2 (should be figure S5)

The statistical analysis of the differential gene expression was performed using DESeq such that figure 8D *vimr2* was identified as a statistically significant gene by this analysis. Full statistical results are provided as supplementary data S4-9. Box plots are now better explained in the legends of S3 (was S2) and S6 (was S5): "box plots defining the distribution of expression levels across these genes in the samples".

6. Please add more details about the method used to facilitate the readers. For example, for the RNA-seq data, please specify which Ensembl release was used and what software was used to plot different plots.

7. Line 561, please cite Ensembl.

6 and 7, thank you for the suggestions, additional information provided and Ensembl cited.

Reviewer 3 Comments for the Author:

1) Overall, I find the experiments to be quite interesting, but there is a general lack of interpretation. I found their conclusions to be wholly unsatisfying and urge the authors to speculate more about what mechanisms might underlie their experimental outcomes. Even the conclusion paragraphs at the end of the results section and throughout the discussion section, the authors are simply re-hashing their results. The text is almost completely descriptive, focused specifically on their experimental outcomes, and there is a lack of broad biological conclusions relating to these outcomes. It is therefore left to the reader to piece together what all of their conclusions might mean. For example, what does it mean that a specific subunit of Cohesin, namely Stag2, specifically impacts Wnt signaling? Why doesn't Stag1 or Rad21 loss lead to the same outcomes?
Do these specific subunits allow Cohesin to take on unique or locus-specific functions, as implied but not directly stated? Even the title and abstract dont sufficiently capture the boarder impacts of the study.

We thank the reviewer for the comment, noting that it's refreshing to be encouraged to speculate! Cohesin-dependent regulation of development is complex, like human cohesinopathies. No single attribute of cohesin can account for the pathobiology of cohesinopathies, so it's no surprise that clear, all-encompassing mechanisms are difficult to extract from animal models of cohesin deficiency.

Morphogenesis in development relies on the complex interaction of cell proliferation with signalling pathways and gene expression, all of which can involve cohesin. Here we show that reducing cohesin (rad21) inclined more towards blocking cell cycle and differentiation and that altering cohesin type (stag2) affected signalling. We think we described this distinction in some detail in the

© 2024. Published by The Company of Biologists under the terms of the Creative Commons Attribution License (https://creativecommons.org/licenses/by/4.0/).

manuscript, and we included work from others on independent functions of cohesin subunits. We added a more in-depth analysis of the *stag2b-/-* single cell RNA-seq data, and that revealed possible mechanisms for the narrower notochord and opened further avenues for discussion (**revised Fig 7, new Fig S11**). The results section has been revised and expanded **(lines 340-385)** and the discussion has been expanded **(line 469-495)** to allay some of the reviewers' concerns.

2) The authors imply that Stag2, and perhaps Stag1, might have function independent of Rad21, and they imply in the introduction and discussion that enhancer regulation (and/or CTCF) might be involved, but the authors do not investigate this hypothesis at all. I suspect based on the results that Stag2 might be particularly important for enhancers that either respond to or stimulate Wnt signaling. What are these enhancers? Could the authors use ATAC-Seq or ChIP- Seq in the tailbud to identify enhancer defects, those that are disrupted from Stag2 loss of function?

We agree performing ATAC-Seq or ChIP-Seq in the tailbud to identify specific enhancer defects associated with Stag2 loss-of-function could identify changes in enhancer regulation. During this study we performed Cut&Run for various histone modifications in the tailbud, aiming to identify altered enhancer activity in *stag2b* mutant tailbuds vs WT. Unfortunately, this approach did not yield the level of resolution necessary to detect significant differences, likely due to the heterogeneity of the tailbud tissue and the dilution of signals from specific cell populations of interest. Because the tailbud is a mix of cell types, it's likely we would need single-cell resolution to effectively discern tissue-specific effects of enhancer activity. Unfortunately single cell chromatin experiments are beyond the scope of the present manuscript, but something we hope to pursue in the future.

3) I found their gene expression analysis to be insufficient. For example, authors state on line 46 that transcriptional changes in rad21 mutants resemble stag2b mutant, and they refer to a PCA plot in Figure 3B. This PCA plot does not provide any comparison of differentially expressed genes. The authors do assess gene expression changes in 3C-E, but they never compare between these groups, as suggested by the text. Such a comparison would be very valuable for assessing outcomes of the mutations. Are differentially expressed genes in one genotype also differentially expressed in another? Do these genes change expression in a similar manner? A thorough gene-by-gene analysis is necessary for this type of comparison rather than a rudimentary PCA.

The data the reviewer wants to see was in the supplemental data in the original version, and in the revised version is now in Figs S4, S5. PCA is shown to represent the separation between the samples to highlight the conclusion that *rad21+/-* and *rad21-/-* are more closely related than to *stag2b-/-* and WT, even though *rad21-/-* is homozygous lethal and the others are viable.

4) In Figure 7B authors use somewhat uncommon and low-resolution method for comparing gene expression patterns between single-cell RNA-Seq datasets. They argue that changes in gene expression are most significant for cells within clusters 9 and 10. It would be much more convincing if the authors sub-sampled populations of cells within each cluster and then applied pseudo-bulk analysis within each sub-population to establish gene expression profiles (simulating replicates), which would then allow them to performed traditional DESeq2 and identify differentially expressed genes. Not only would this provide an orthogonal means to assess gene expression differences, but it would also allow the researchers to identify the most differentially expressed genes, and then assess gene ontology or pathway analysis.

We thank the reviewer for the suggestions. Deeper analysis of the scRNA-seq data did indeed reveal some aspects of the biology that were not previously apparent. We have **revised Figure 7** dropping the Augur analysis and instead applying gene set enrichment analysis for molecular function on differentially expressed genes on a per cluster basis or also known as pseudo-bulk. We split the sample into 3 pseudo-replicates and performed DESeq2 analysis on pseudo bulk data. We found slightly more genes (n=1125) significantly differentially expressed using pseudo-replicates compared to the more common Wilcoxon rank sum test (n=695). We found 356 out of 695 or a 51% overlap between Wilcoxon rank sum test and pseudo-replicate analysis in the notochord cluster (see table for_review_notochord_pydeseq_wilcox.xlsx) We decided to not use pseudo-replicates

© 2024. Published by The Company of Biologists under the terms of the Creative Commons Attribution License (https://creativecommons.org/licenses/by/4.0/).

since this might artificially inflate the number of DEGs. Instead we used the more common Wilcoxon rank sum test comparing wild type and *stag2b*$^{-/-}$ mutant tailbud single cell clusters. We are thankful for the suggestion to use pathway analysis such as gene set enrichment on molecular function or KEGG pathways on DEGs identified by pseudo bulk methods. We **updated figure 7C** and provide a **new figure S11** with gene set enrichment analysis plots for NMPs and notochord clusters respectively. The analyses are supported by **new supplementary data S7-9**. In the case of the NMP cluster we identified pathways such as EMT transition and stem cell maintenance, which are downstream of Wnt signalling and downregulated in *stag2b*$^{-/-}$ embryos. This analysis further supports the rationale to rescue the effects of *stag2b*$^{-/-}$ by stimulating Wnt using BIO. In addition the analysis of the notochord cluster comparing *stag2b*$^{-/-}$ and wild type embryos shows subtle changes with loss of notochord identity and an increase of hypochord marking cells which could at least partially explain the thinner notochords in *stag2b*$^{-/-}$ mutant embryos. In summary we **revised Fig 7, provide a new Fig S11**, revised the results **(lines 340-385)** and the discussion **(line 469-495)** in response to this point.

5) Authors rely pretty heavily on a single method for manipulating wnt signaling. The paper would be much stronger if there were orthogonal strategies to disrupt wnt signaling. I could imagine inhibiting wnt or using a morpholino to inhibit expression of wnt8. The expectation is that these inhibition experiments would phenocopy Stag2 mutants, or perhaps the stag2 deficient embryos would be more sensitive to wnt inhibition.

Inhibition of Wnt is an interesting suggestion but is practically very difficult to do - Wnt signalling is essential for proper embryo development, so interference at early developmental stages are likely to mess up the embryos. Previous experiments blocking Wnt8 using a morpholino, or a Wnt8 deficiency, produces fairly extreme phenotypes that do not resemble *stag2b-/-* : Figure below from Levken et a (2001) PMID: 11703928

NOTE: Figure provided for reviewer has been removed. It showed 7 from **Lekven, A. C., Thorpe, C. J., Waxman, J. S. and Moon, R. T.** (2001). Zebrafish wnt8 encodes two wnt8 proteins on a bicistronic transcript and is required for mesoderm and neurectoderm patterning. *Dev. Cell* **1**, 103-114. Doi:10.1016/s1534-5807(01)00007-7. We have removed unpublished data that had been provided for the referees in confidence.

In contrast, *stag2-/-* embryos look almost normal, and are viable to adulthood. It is possible that they could be more sensitive to very low doses of Wnt8 morpholino. However, we previously published a paper where we show multiple methods of Wnt stimulation in wild type and cohesin-mutant backgrounds in zebrafish and cell lines (Chin et al, eLife 2020 PMC7746233), moreover, other research teams have confirmed the reproducibility of Wnt manipulation using various compounds on cohesin-deficient zebrafish (PMC6601076, PMC8615450) human cells (PMC11011321) and Drosophila (PMC7889653), so we don't consider it necessary to perform extra controls that reinforce our choice of method to stimulate Wnt signalling in this particular manuscript.

Second decision letter

MS ID#: DEVELOP/2023/202593

MS TITLE: Cohesin composition and dosage independently affect early development in zebrafish

AUTHORS: Anastasia A Labudina, Michael Meier, Gregory Gimenez, David Tatarakis, Sarada Ketharnathan, Bridget Mackie, Thomas F Schilling, Jisha Antony, and Julia A Horsfield

I have now received two referees reports on the above manuscript, and have reached a decision. The referees' comments are appended below, or you can access them online: please go to BenchPress and click on the 'Manuscripts with Decisions' queue in the Author Area.

© 2024. Published by The Company of Biologists under the terms of the Creative Commons Attribution License (https://creativecommons.org/licenses/by/4.0/).

The overall evaluation is positive and there are just a few issues from one referee to address before we proceed to publication. Please attend to all of the reviewers' comments in your revised manuscript and detail them in your point-by-point response. If you do not agree with any of their criticisms or suggestions explain clearly why this is so.

Reviewer 2

Advance summary and potential significance to field

Cohesin, a multi-protein complex, plays critical roles in cell proliferation and gene expression. A significant amount of effort has been put into understanding the molecular basis underlying the regulation of chromatin structure by this complex. However, much is known about the complicated relationship between the subunits of the complex, especially in a developmental context. In this manuscript, Labudina and colleagues aim to understand the developmental consequences when different cohesion subunits are mutated.

The authors analyzed the morphological and molecular phenotype of rad21-/-, rad21+/-, stag2b-/-, and stag1b-/-;stag2b-/-. They found that mutations of stag2b and rad21, which may represent altering composition versus quantity of cohesin, have different developmental consequences. They discovered that the notochord thickness is significantly narrower in stag2b mutants and further revealed that Wnt signaling is likely underlying this narrow notochord phenotype. Interestingly, they found that the stag2b mutation also rescues the narrow notochord phenotype. These findings address the molecular and developmental functions of different cohesin subunits, which are informative to the developmental and stem cell community.

Comments for the author

Major:
“Stag1b and Stag2b are the main functional Stags in zebrafish” is overstated. This statement means that all the other Stags are not functionally important during zebrafish development, which is not supported by their results.
Firstly, the new statement “stag2a mRNA is barely present” is misleading. Although “stag2a is the least expressed of all the stag genes” during late embryogenesis, stag2a is expressed in early embryos (Ketharnathan et al., 2020). In fact, “At early gastrula stages, … stag2a expression was noticeably reduced compared with stag1a/b and stag2b.” This may suggest that stag2a plays a role during early embryogenesis.
Secondly, their lab has previously tried to generate stag2a mutants but failed: “No germline mutations could be recovered in stag2a despite evaluating multiple guide RNAs.” (Ketharnathan et al., 2020). This may suggest that stag2a is indispensable for zebrafish development.
Thirdly, while the lethality phenotype of stag1b-/-;stag2b-/- mutants does underscore the importance of Stag1b and Stag2b, it's important to exercise caution in concluding that Stag2a is not functional. Without the stag2a mutant, definitive statements about its functionality are premature.
In conclusion, I strongly suggest the authors to reconsider their statement. For instance, a more appropriate subtitle could be, “Loss of zebrafish Stag1b and Stag2b phenocopies the null cohesin mutation rad21”.
Minor: Please cite the software and tools used for plotting.

My other concerns have been addressed adequately. Thanks.

Reviewer 3

Advance summary and potential significance to field

Although reviewers did not take all of my suggestions, they have addressed many concerns raised by myself and other reviewers. I am now satisfied with their responses and find the manuscript suitable for publication.

© 2024. Published by The Company of Biologists under the terms of the Creative Commons Attribution License (https://creativecommons.org/licenses/by/4.0/).

- Patrick Murphy

Comments for the author

None.

Second revision

Author response to reviewers' comments

Reviewer 2 suggestion:
In conclusion, I strongly suggest the authors to reconsider their statement. For instance, a more appropriate subtitle could be, “Loss of zebrafish Stag1b and Stag2b phenocopies the null cohesin mutation rad21”.

Response:
We accept the reviewer’s standpoint and have changed the subtitle (line 170) and the title to Fig. 1 along the lines of their suggestion: “Combined loss of zebrafish Stag1b and Stag2b phenocopies the null cohesin mutation rad21”

We deleted the sentence at line 202 referring to Stag2a’s expression and modified the concluding sentence to read “Our results suggest that Stag1b and Stag2b cannot be compensated by Stag1a and Stag2a proteins in zebrafish.”

Reviewer 2 suggestion:
Minor: Please cite the software and tools used for plotting.

Response:
We added this reference at line 642: “R package ggplot2 was used for data visualisation (Wickham et al., 2016).”

H. Wickham, W. Chang, M. H. Wickham, Package ‘ggplot2’. Create elegant data visualisations using the grammar of graphics. Version 2, 1-189 (2016).

Third decision letter

MS ID#: DEVELOP/2023/202593

MS TITLE: Cohesin composition and dosage independently affect early development in zebrafish

AUTHORS: Anastasia A Labudina, Michael Meier, Gregory Gimenez, David Tatarakis, Sarada Ketharnathan, Bridget Mackie, Thomas F Schilling, Jisha Antony, and Julia A Horsfield

I am happy to tell you that your manuscript has been accepted for publication in Development, pending our standard publication integrity checks.

© 2024. Published by The Company of Biologists under the terms of the Creative Commons Attribution License (https://creativecommons.org/licenses/by/4.0/).
